# On the Influence of Climate in Pulmonary Consumption

**Published:** 1817-06

**Authors:** Thomas Sutton


*For the London Medical and Physical Journal.*

### *On the Influence of Climate in Pulmonary Consumption;* by THOMAS SUTTON, M.D.

I AM not aware that I promised not to communicate any thing further on pulmonary consumption, that would interfere with Dr. Buxton's opinions. I certainly wished to discuss the subject, in any thing I might in future have to communicate, without any personal allusion to him. This I did, as I conceived, in my two last letters, published in the Medical and Physical Journal. I however, in fact, meant meant the last letter to be a postscript to the former, and to be published in the same Number of your Journal, which the dates of them will shew. The last, it seems, was mislaid, about which I never enquired, and for many months past I have entertained no expectation of seeing it published. Dr. Buxton, it appears, has thought that this letter required his animadversions, as offending against his opinions. But he may very readily see that I had very little anxiety to do this, having suffered the letter to remain unpublished upwards of a year; and I should certainly have never enquired the reason of its not being printed, although it never had appeared. It must not, however, be supposed, that I did not consider the returns, which are the foundation of the last paper, to be important documents;—but, as much had been said by me in support of the opinions I have maintained on consumption, I considered it not to be amiss to remain a little while quiet on the subject.

Dr. Buxton has, it seems, taken some pains, in his last letter, to shew that I have inferred the frequency of consumption in Malta "by a strange mode of proof." I am, however, quite content, that my inference can be very well supported at present. The doctor must also be well acquainted, that we do not agree about the actual causes of consumption so entirely as to be likely to go the same way to work in forming our conclusions.

I shall add very little further remark on Dr. Buxton's paper; but, as he has made some observations on one of the returns, I shall, sooner than I intended, take up the consideration of three documents of this kind, from three military stations, that I have been favoured with from Sir James M'Grigor.

The object of much of the discussion that has passed between Dr. Buxton and myself, has been, to ascertain the frequency of consumption in various parts of the world. In so far as the Mediterranean and West Indies are concerned, the returns I at present possess will throw great light on the question; and I think they may afford a good guide to infer, to what extent consumption exists elsewhere, in similar situations.

I have first to remark, that the sort of estimate we make of the frequency of this disease, in comparing it with the general mortality, is most fallacious and uncertain. A Table of these returns will immediately demonstrate this.

The Malta return includes only one year—the other returns include two years. In the Malta return, the number of troops are set down as varying every month. The highest number in any one month is 4204; the next lower number is 3800; and the lowest number in any one month is 2747. Setting down, therefore, 3500 as the number during the whole of the year, the exact average is considerably exceeded.

TABLE I.

| | Year. | No. of Troops. | Total of Deaths. | Deaths by Consumption. |
|---|---|---|---|---|
| Malta | 1814 | 3500 | 51 | 10 |
| Gibraltar | 1813 | 3810 | 441 | 17 |
| | 1814 | 4304 | 177 | 15 |
| Jamaica | 1813 | 4292 | 370 | 33 |
| | 1814 | 4197 | 314 | 38 |

In comparing the number of troops given above in Malta, with the number of deaths by consumption, with the second line of figures, in respect to the number of troops in Gibraltar, with the deaths also by consumption, it must immediately appear that the deaths by this disease in Malta are less than those in Gibraltar, and so on with the rest. But, if we take an estimate of the number of deaths by consumption, averaging them by the total number of deaths in each place, we shall find that they will be as one to five dying in consumption in Malta, and as one to twenty-six dying of consumption at Gibraltar, &c. It is, therefore, concluded, by our present method of estimate, that the greatest mortality in consumption must be in Malta, which, according to the Table, is evidently not the case. This sort of measure of the mortality of consumption is also adopted to shew its relative frequency, in various parts of the world, in comparison with Great Britain, where it is supposed to occasion the death of one in five.

Let us now make the supposition, that some grievous disease had attacked the inhabitants of this happy island, and had swept off 500,000 more of its inhabitants than usually die in one year. If we were to take such a period as this to form our estimate of the prevalence of consumption, it would come out in proof, that much fewer in proportion die of that disease than we at present estimate. Are we, then, to take this period as a proper time to arrive at the average prevalence of consumption? I may say, certainly not. If so, when we compare our average number of deaths by consumption with the general mortality, and allow the same comparison to be made with other countries, it is strange that it never has occurred to compare the general mortality in other countries with our own; for, unless we do so, our measure is quite vague and indeterminate. This has led us into constant error. It is not, therefore, arriving at the truth, by enquiring whether one in five, one in ten, or one in in twenty, die of consumption, in any particular country, in comparison with what happens of the same kind in our own, unless we first compare the average mortality of each country. Where this exists proportionably equal with our own, we shall be able to infer the relative mortality in consumption; but, under no other circumstances, by our present mode of estimate. For, although I doubt not that consumption is somewhat increased, by the great mortality that occurs in any certain country or station, yet it by no means follows the rule of increase or decrease in an equal proportion to the healthiness or unhealthiness of a place. When, therefore, the general mortality in different countries is different, the way to arrive at a proper conclusion, respecting the prevalence of consumption, is, by an estimate of the number of deaths by that disease in proportion to the number of inhabitants; or, where it can be arrived at, the proportion between the mortality in different countries, by bringing them to some common standard, and thence deducing the comparison.

I shall presently make use of the returns, to shew the relative prevalence of consumption in Malta, Gibraltar, and Jamaica, by the first-stated means, being acquainted with the number of subjects in each of these military stations: and, by way of comparison, I shall give a table of the results, by our usual mode of reckoning, respecting the prevalence of this disease, in the same places.

TABLE II.

| | Year. | Total of Deaths. | Deaths by Consumption. | Average of Deaths by Consumption to the whole Mortality. |
|---|---|---|---|---|
| Malta | 1814 | 51 | 10 | One in 5 |
| Gibraltar | 1813 | 441 | 17 | 26 |
| | 1814 | 177 | 15 | 12 nearly |
| Jamaica | 1813 | 370 | 33 | 11 |
| | 1814 | 314 | 38 | 9 nearly. |

This table not only evidences the uncertain measure we have adopted to arrive at the relative mortality of consumption, by placing it at more or less, according to the total mortality; but actually shews Malta to be the greater sufferer by that disease, which, from an inspection of the first table, it evidently is not. The actual relative state of the prevalence of consumption in the three places noted, is according to the returns, as follows:

TABLE III.

| | Year. | No. of Troops. | Of these died of Consumption in one Year. | Number out of which one Person may be expected to die of Consumption yearly. |
|---|---|---|---|---|
| Malta | 1814 | 3500 | 10 | 350 |
| Gibraltar | 1813 | 3810 | 17 | 206 |
| | 1814 | 4304 | 15 | 280 |
| Jamaica | 1813 | 4292 | 33 | 133 |
| | 1814 | 4197 | 38 | 110 |

Hence an evident result is obtained by the last Table of a very different kind to that afforded by founding the calculation on the existing general mortality of a place or country. In a confined circle, the plan adopted by the last table would be evidently the familiar method of enquiry. If we wish to ascertain the prevalence of consumption in a family, our chief objects would be to ask, how many died of the disease, and of how many the family consisted? We should not think it connected with this specific enquiry, to ascertain, for the sake of comparison, the number that died of other diseases. But, if we found the consumptive cases to exceed what generally happened in other families of a similar number, we should consider ourselves qualified to form our conclusions respecting the disposition to the disease, and its frequency in that particular family. This could not be so well done in a large and extended community. To obtain, therefore, some notion of the comparative frequency of consumption in various places, recourse has been had to a comparison of the fatal cases of this disease with the total mortality, never adverting to the circumstance that the former disease has no relative proportion of increase in various countries with the total deaths that occur among their inhabitants.

The usual mode of calculating consumption is even erroneous, when it appears to lead directly to similar results. For instance, according to the Malta return, one death in five has happened by consumption: hence it might be concluded, that the mortality in consumption in this country, bearing the same proportion, was equal to that of the same disease in Malta. But this may, nevertheless, not be the case. For, if it is proved that the total mortality in Malta is greater than as it occurs in England, and that the mortality by consumption bears the same proportion to the total deaths in each place, it must immediately appear evident, that the mortality by consumption in Malta must be greater than as it exists with us. This may readily be demonstrated by a measure that is already prepared for me. Sir Gilbert Blane, in his paper on the Health of the Navy, published in the Medico-Chirurgical Transactions, vol. vi. has endeavoured to ascertain the relative mortality in the service of our navy, and as it occurs in this country in civil life; and, after making very liberal allowances on the part of the latter mortality, so as to make it as high as he well could, he infers, that, of persons of the same general range of age with sailors, one in eighty dies in civil life annually. Now, the ages of soldiers and sailors are similar; therefore I may, without hesitation, assume his calculation, as applied to the army, in what I may afterwards have to advance on the subject of this inference. According to our estimate in this country, one in five dies of consumption; then, on multiplying eighty by five, we arrive at the number out of which one will die annually by consumption, out of those who are within the usual range of the age of soldiers and sailors; that is, of such as are from twenty to forty, which comprehends the great bulk of them,—and within these ages most of the consumptive may be included. The sum thus obtained will be 400. If, therefore, the same proportion of mortality by consumption* happen in Malta, among the soldiers,

---

* This may seem to be erroneously assumed, as one in five only dies of consumption, comparing the relative number of deaths by this disease as to frequency with the deaths of all ages. But I am now speaking of the military, and take Sir James M'Grigor's observation as a standard, who asserts that deaths by consumption among the troops in Great Britain amount to one in five of the deaths by other diseases. On a first consideration, we might have expected a different proportion; but, as this is stated as a matter of fact, collected from various observations, we must take it as such, and enquire how this is likely to happen. Though the military are of the general consumptive age, yet it must be remarked, that none of infirm health, or consumptive, are admitted to be soldiers; and it is to be observed, that a more than equal share of those who die of consumption are of the class of those who cannot be admitted into the army. Next it may be observed, that women are, upon the whole, more liable to consumption than men. These two causes appear to effect an equality between the whole of the deaths in civil life, compared with those by consumption, and such as happen among the military in this country, in the total, compared with the same. Had Dr. Buxton added these considerations to those which he thought weighed so much in his favour, to diminish the real frequency of consumption as stated in his last letter, against the evidence of the returns, together with the decided matter-of-fact statements of Sir James M'Grigor, he would, soldiers, then, by multiplying 400 by the number of deaths occurring in Malta by consumption in one year, we shall arrive at a knowledge of the number of persons included in that return, if the disease prevails in equal proportion there as it is acknowledged to exist here. That number would be 4000. In so far, therefore, as this number exceeds the amount of the returned military in Malta, (they being stated at 3500,) which it may be observed to do considerably, to such extent does consumption prevail more in Malta than in this country; that is to say, at least in the ratio of 40 for the latter, to 35 of the former, and, by multiplying each number by 10, bring the amount of each to 400, out of which one consumptive patient is expected, as stated, to die annually in this country; and 350, out of which one consumptive patient dies at Malta.

---

would, with difficulty, have arrived at the conclusions which he is still willing to maintain.

Dr. Buxton, I find, also thinks that I have maintained my opinions, in opposition to his, "by separating particular passages, and by detaching them from their situation, given them a force which they do not possess." I really believe he thinks so; but, if the reader of this note can confide in my assertion, I will assure him, that I have not employed myself in any such inferior work. I have laid always more stress upon absolute facts in the writings I have alluded to, than on opinion, or on opinions, inferred from fact. I have, therefore, treated opinions with less importance than facts; and have paid little deference to one part of an author's book, and placed great stress on another. Whether I have taken the right way to arrive at truth on the subject, must be submitted to the judgment of others. Lastly, Dr. Buxton seems to consider it to be an offence, that I have opposed his opinions; but, as he has taken a prominent station in advocating opinions, which, though generally entertained, to the best of my judgment, I neither thought just or useful, he has hazarded the same opposition that others in a similar station might encounter. In so far as his opinions are just, they will have their due weight; opposition can only, in such a case, exalt their merit and that of their supporter, and the opposer gain nothing but discredit. If Dr. Buxton thinks himself in the happy situation of advocating indubitably useful opinions, founded upon a just consideration of all the circumstances connected with them, he ought to hail me as his best friend, and congratulate himself whenever he finds I take the trouble to draw him or his opinions on consumption before the public. Of this, however, he may be assured, that there is not a man existing against whom I can have less personal enmity than against him.—So far I thought it necessary to say in a note, that I might not interrupt the continuity of my present paper.

It

It will be seen by the Gibraltar return, that consumption prevails there to a considerable extent. Yet Sir James M'Grigor has made a statement, in his paper on Diseases of the Army (inserted in the 6th volume of Medical and Chirurgical Transactions), calculated to produce a strong impression, that consumption is a very rare disease in Spain. The favourable opportunity he seemed to have possessed of making observations, and on apparently so large a scale, must have given considerable weight to them. But, it may be asked, how can this disease exist to a considerable degree in Gibraltar, and rarely occur in other parts of Spain? To solve this difficulty, we might be induced to investigate local circumstances; but this great discrepancy only exists in consequence of the mode of calculation. It is very true, that the inference must be such as Sir James M'Grigor has made, in comparison with the total number of deaths. But it is also true, that a state of active warfare very much augments the general mortality of those concerned in it. As, therefore, this augmented mortality must be an uncertain measure of the mortality of the same number of persons, under different circumstances, and in civil employs, so also it is an imperfect measure of the frequency of consumption, that neither becomes proportionally frequent with either an accidental increase of mortality, nor bears an equal ratio with the mortality produced by the causes of general sickness, or unhealthiness, existing in many countries or climates. But, when this statement of Sir James M'Grigor is placed on the footing of a comparison of the mortality in consumption with the number of persons comprehended in his statement, it will readily appear, that no such inference as has been drawn from it can be maintained. It will be evident that some of my information, to arrive at this conclusion, is imperfect; but it will, I trust, be found to be of such a character as to be competent to the intended object.

The number 400 has been before stated to be that out of which one individual, from 20 to 40, dies of consumption, in this country. If we, therefore, multiply this number by the number of individuals who have died of consumption in the Peninsular army, in so far as this product exceeds, or is equal, or falls short, of the number of individuals who composed that army,—to such extent did consumption prevail more, or equally, or less than it is supposed to do in Great Britain. Sir James M'Grigor has acquainted us that 279 persons died of consumption in all the British hospitals in Spain, during the period he comprehends in his account. This period is two years and a half, and will, by this calculation, make the average number of British troops during the the whole of it to amount to upwards of 44,000, supposing the deaths by consumption to be equal in frequency to what occurs in this country. But, in Sir James M'Grigor's tables are also placed more than one-fourth of the numbers that have been received into the different hospitals under the title of transferred, and are heard no more of, excepting of 4586 comprehended under that head, who were invalided and sent to England, as no longer fit for active service in the field, or for the recovery of their health. In one part of Sir James M'Grigor's report, we likewise find that consumptive cases were set down to be sent to England, " resting some hopes of benefit from a sea voyage," and, as I suppose, included in the 4586. Of these, if we assume that forty died of consumption, the whole force of British in the Peninsula, for the whole period of Sir James's superintendance of the army hospitals, must, by this calculation, amount to upwards of 50,000 men, which I am assured it did not; or consumption must prevail more in the Peninsular army than in Great Britain. Whether, however, my data are accurate or not, it must be very evident, that consumption prevails in Spain in a much greater degree than might be concluded from Sir James M'Grigor's report; and, on an exact view of all particulars, it may be found to exist in the same proportion as at Gibraltar.

Though the most accurate method of estimating the relative prevalence of consumption must be by comparing the number of deaths in each country by that disease, with the total number of the different inhabitants of each, yet this would be very troublesome to execute, and liable also to many errors, in large communities. From what has been said, therefore, an artificial means might be adopted, which would insure a tolerably near approximation to the truth. This may consist of the numbers that die annually in different countries under any of the ordinary circumstances of mortality, with the relative number of deaths annually by consumption in each, together with an estimate of the number of inhabitants from which one individual dies yearly in each country. Thus, for instance, if, in any one place, it should be found that one in nine, of the total mortality, dies of consumption, but that the mortality, in respect to the inhabitants, is 1 in 60, and we state the mortality of the inhabitants in our country, for the sake of hypothesis, at 1 in 90, then the mortality in such a country by consumption, in comparison with our own, would be as 6 to 5. Again, if the mortality should be found in any place at 30, that is to say, 1 in 30 dying annually, and the ratio of consumption to the general mortality shall be as 1 in 15, stating also the mortality, mortality, as above, in this country at 90, and the deaths by consumption as one in five, then it will be perceived to be equal in both countries, instead of the great disproportion which would be made to exist in the frequency of this disease, by the common mode of calculation, under such circumstances.

Such, then, must be the proceeding to obtain proper conclusions, by the general mortality respecting the comparative frequency of consumption in various countries; for a mere comparison of the general mortality, compared with the number dying of consumption, can seldom lead to accurate conclusions—and never, unless the total mortality shall be found equal in each of the countries that becomes the subject of the comparison. But the true and accurate mode is to compare the single disease of consumption with the whole of the population of each country, or with such a proportion of the population as may comparatively be supposed to represent the whole. Thus, in a district of 4000 inhabitants, if we ascertain the numbers that die of consumption annually among them, in comparison with the whole, we attain to the object sought for, which may be inferred to represent the mortality by this disease, through the adjoining communities, under similar circumstances. This will fairly serve us to compare with the mortality, by consumption, so acquired in any distant country. The invention I have spoken of, when the data can be fixed, may serve also as a substitute for this, keeping, however, always in mind, that the total mortality, in different countries, can very seldom be an accurate measure of the relative prevalence of consumption.

By attention to the above considerations, and the methods of proceeding pointed out, it will evidently appear, by the military returns in my possession, that more mortality exists in Malta and Gibraltar than in this country; and we may with every probability infer, that it exists generally on the Mediterranean shores, and countries adjoining, in the same proportion, as well as in climates similar or approximating to them. From similar evidence we may also perceive, that this disease prevails in a still greater proportion in the West Indies than in the countries just alluded to.

An enquiry respecting the relative mortality by consumption in various countries and climates, may certainly be viewed as a proper exercise of curiosity. But, if it had merely been confined to that object, it would probably not have gained my particular attention. This enquiry, conducted as it has hitherto been, has been made use of for much more important purposes, that of giving a material direction to the treatment of pulmonary consumption, grounded grounded on a fallacious estimate of its frequency in various climates. A mode of treatment of consumption has, by this means, been supported, which is most seriously wrong in many particulars, and has rendered the disease, I am certain, comparatively much more fatal. This erroneous statement, however, has not only produced the disadvantages mentioned; but has laid the foundation for opinions, which are acted upon to foster and promote the disease in instances in which it might not have otherwise occurred.

With such conviction, I began the inquiry respecting the comparative mortality by this disorder in various countries, with few means to guide me, but promoting and seeking for information in every direction that occurred to me. That I have not been supplied with more ample information, is not my fault; but, of that which I possess, I have enough to point out the proper way of enquiry on this question (rendered highly important by the use that has been made of it), to solve much of the difficulties connected with it; and to demonstrate the existence of consumption in a much greater degree in countries heretofore said to be comparatively free from it, than as it is allowed to occur in Great Britain.

*Croom's Hill, Greenwich;*

